# Nanoporous Gold as a VOC Sensor, Based on Nanoscale Electrical Phenomena and Convolutional Neural Networks

**DOI:** 10.3390/s20102851

**Published:** 2020-05-17

**Authors:** Timothy S.B. Wong, Roger Newman

**Affiliations:** Department of Chemical Engineering and Applied Chemistry, University of Toronto, 200 College Street, Toronto, ON M5S 3E5, Canada; roger.newman@utoronto.ca

**Keywords:** volatile organic compound, nanomaterials, nanoporous, frequency response, convolutional neural network

## Abstract

Volatile organic compounds (VOCs) are prevalent in daily life, from the lab environment to industrial applications, providing tremendous functionality but also posing significant health risk. Moreover, individual VOCs have individual risks associated with them, making classification and sensing of a broad range of VOCs important. This work details the application of electrochemically dealloyed nanoporous gold (NPG) as a VOC sensor through measurements of the complex electrical frequency response of NPG. By leveraging the effects of adsorption and capillary condensation on the electrical properties of NPG itself, classification and regression is possible. Due to the complex nonlinearities, classification and regression are done through the use of a convolutional neural network. This work also establishes key strategies for improving the performance of NPG, both in sensitivity and selectivity. This is achieved by tuning the electrochemical dealloying process through manipulations of the starting alloy and through functionalization with 1-dodecanethiol.

## 1. Introduction

Volatile organic compounds (VOCs) are universally present in the modern world. From their usage in household staples, such as laundry detergent, to their applications in industry, in the form of oil and gas, VOCs are a critical pillar on which modern society rests [1,2]. Due to their high vapor pressure, VOCs inevitably evaporate into surrounding environment, necessitating reliable gas phase sensing [1,3]. While often discussed as a single class of chemicals, individual VOCs pose their own unique health and safety risks. These risks are further amplified by the rapid diffusion in the gas phase. Once evaporated, VOCs can quickly pose significant health and safety complications over a large area [4,5].

One practical example of the importance in identifying and sensing individual VOCs is the discrimination between ethanol and methanol; methanol poses significantly greater health risk than ethanol. Inhalation of methanol can lead to damage to the nervous system and death, whereas ethanol is regularly safely consumed in beverages [6,7]. These significant risks underscore the need for sensor technology to discriminate and sense individual VOCs in the gas phase. Existing commercial VOC sensors do not meet these requirements, eschewing selective sensing for a broad approach where the total concentration of VOCs is reported [8,9,10]. This approach is wholly insufficient for modern applications due to the unique nature of individual VOCs. Current sensor research is focused on implementing novel materials for highly specific sensors of VOCs, such as acetone, ethanol, and methanol [7,11,12,13]. However very little work has been done in applying novel materials in creating a selective sensor that is capable of simultaneously classifying and quantifying a range of VOCs.

Nanoporous gold (NPG) is an exciting new nanomaterial for sensor applications in three key aspects. First is its nanoporous nature, which allows for sensing mechanisms to leverage unique electronic and physical nanoscale phenomenon. Furthermore, the large surface area of nanoporous materials inherently amplifies the sensor response [14,15,16]. This amplification allows for normally undetectable changes to become measurable. Second is its relative ease of fabrication in comparison to other nanomaterials. While many other sensors leveraging novel nanomaterials require complex and highly sensitive fabrication processes, NPG can controllably and easily be fabricated through electrochemical dealloying; where a controlled electrochemical bias is applied to an Au alloy, causing the dissolution of a sacrificial component and the surface diffusion of gold atoms resulting in a nanoporous structure [17]. Finally, NPG offers sensor robustness owing to the chemical stability of the Au surface layer. These characteristics of NPG have resulted in a variety of novel sensors applications, such as an electrochemical sensor of viruses or as a photonic sensor for heavy metals [16,17,18]. One notable sensor area where minimal work has been done in leveraging the unique properties of NPG is in gas sensing [15,19,20]. Furthermore, the methods of data analysis in this space have also remained rudimentary.

This work builds on previous research where NPG was leveraged as a humidity sensor by measuring the impedance response and applies NPG as a selective sensor for VOC through the measurement of the complex frequency response spectrum [20]. We propose two mechanisms by which the frequency response changes in response to changes in the gas phase concentration of VOCs: surface adsorption induced changes in resistance, and condensation induced changes in capacitance. We measure the changes in frequency response in five model compounds (acetone, ethanol, hexane, methanol and water). These five model compounds are selected to represent a broad cross section of common VOCs and water. We utilize the frequency response measurements of each compound to train a convolutional neural network (CNN) to classify frequency responses into one of the five compounds. We subsequently present unique CNNs to quantify the concentration of each compound. We evaluate the performance of each trained neural network and implement material and surface improvements to achieve a novel, selective VOC sensor based on NPG.

## 2. Materials and Methods

### 2.1. Materials and Sample Preparation

Ag_77_Au_23_ (at%) used in electrochemical dealloying to fabricate NPG was purchased from Goodfellow (Cambridge, UK) and rolled to 100 µm in thickness. Ag_77_Au_23_Pt_3_ (at%) alloys were obtained as a cold-rolled 200 µm sheet from Ames National Laboratory—US Department of Energy, Iowa and subsequently rolled to 100 µm in thickness. Samples roughly 10 mm × 5 mm in size were cut from the rolled AuAg sheet and annealed at 900 °C for 3 h under an Ar/H_2_ (2.5% H_2_) gas flow in a tube furnace. AuAgPt samples of a similar size were cut and annealed at 1050 °C for 12 h under the same atmosphere; annealing relieved alloy stress and reduced silver oxide. Small end sections of copper wire (~15 cm in length) were stripped and soldered to the annealed samples. The solder junction, any remaining exposed copper, and the backside of the alloy were covered with lacquer (SPI Microshield) and allowed to dry overnight. This lacquering process was repeated to prevent any stray contact during the dealloying process. The exposed surface area was measured using a Zeiss Stemi−2000 C optical microscope.

Dealloying solutions were prepared with Analar grade concentrated HClO_4_ (62%, Alfa Aesar) with deionized water at a concentration of 0.5 M. Before dealloying, these solutions were deaerated by purging with N_2_.

### 2.2. Electrochemcial Dealloying

Electrochemical dealloying was carried out using a Gamry Reference 600 Potentiostat in deaerated 0.5 M HClO_4_ solutions. NPG samples were dealloyed by applying a fixed potential, 550 mV (MSE, 640 mV vs. Standard Hydrogen Electrode), with a Pt wire counter electrode until 25 C/cm^2^ of charge density had passed. Based on previous work in our group, the expected dealloying depth is approximately 50 µm [21]. After dealloying, lacquer was stripped using SPI Microshield Remover. Samples were immersed in deionized water for 24 h to allow for residual solution to diffuse out and then allowed to dry under a flow of dry air.

### 2.3. Material Characterization

After drying, characterization on samples was performed in a Hitachi S5200 (SEM) Scanning Electron Microscope in secondary electron mode at an acceleration voltage of 20.0 kV and a beam current of 20 µA.

### 2.4. Functionalization

Some NPG samples were functionalized by a method adapted from Hakamada et al. [22]. In summary, samples were immersed in a 10 mM thiol solution for 3 h. After immersion samples were thrice rinsed with ethanol and allowed to dry overnight. 10 mM thiol solutions were prepared from 1-dodecanethiol (Alfa Aesar) and ethanol.

### 2.5. Electrical Measurement

Electrical measurements of NPG were performed using a four-point probe configuration due to the small impedance of NPG; the current delivery and voltage measurement were performed by separate pairs of probes. The output voltage signal was amplified by an AD620 instrumentation amplifier with a 1 kΩ bias resistor. A reference signal was captured across a 1 Ω series resistor. The input and amplified response signal were analyzed by a frequency response analyzer CV 2001 (Capcis Voltech) and the frequency response was recorded via RS-232 connection to a computer. The frequency response between 1 Hz and 100 kHz was recorded at 60 equally log-spaced points.

### 2.6. Controlling VOC Concentration

VOC concentration was controlled within a polypropylene chamber, approximately 100 mL in size. Two holes were cut into the chamber to allow for inflow and to pass electrical leads connecting the four-point probe. Inflow was maintained at a rate 500 mL/min. Air was first dried by passing through a desiccating column filled with indicator Drierite. VOC concentration was modulated by the volume controlled mixing of a bypass stream of dry air and a separate volatile saturated stream. The system was allowed to equilibrate for 5 min after volumetric flow changes were made before electronic measurements were taken.

### 2.7. Data Processing and Analysis

The measured frequency response was converted into complex impedance through comparison with the reference resistor. The responses were subsequently filtered through a discrete median filter (window size = 3), to minimize the effect of extreme outliers.

Neural networks were trained using a random subset of 80% of the gathered data with the remainder utilized for model evaluation. The training set was used to scale the data to a standard deviation of 1 and an average value of 0. Training was performed by the back propagation of loss, and model parameters were optimized by stochastic gradient descent. This process was repeated 5 times for each model to achieve a complete and objective understanding of model performance, with average accuracy and resolution reported. Models were trained over 200 epochs with a learning rate of 0.01. Data preprocessing, model training, and evaluation were done in Python3 using the NumPy, sklearn, SciPy, and PyTorch libraries.

## 3. Results and Discussion

### 3.1. Material Characterization

**Figure 1:**
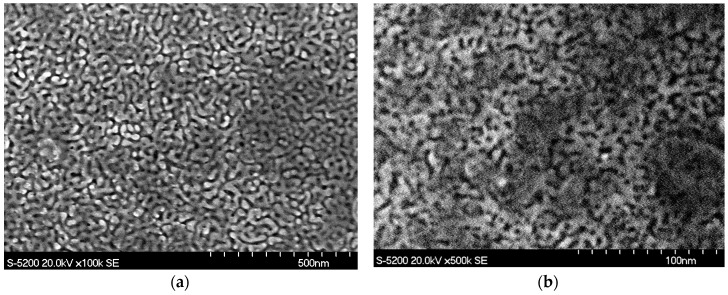
SEM micrographs of electrochemically dealloyed samples: (**a**) Ag_77_Au_20_ (NPG—nanoporous gold); (**b**) Ag_77_Au_23_Pt_3_ (NPG-Pt).

Upon application of the electrochemical bias, color change was quickly observed; with samples turning from silver to a brown-gold color. Dealloying current rapidly stabilized between 5–6 mA/cm^2^ for AuAg samples and 3–4 mA/cm^2^ for AuAgPt samples. Characterization by SEM (Figure 1) qualitatively confirmed published results on the ligament and pore size published by our group [21,23]. This study reported a mean ligament width of ~14 nm with a mean pore size of ~17 nm for electrochemically dealloyed AuAg (NPG) and the mean ligament width was observed to be ~4 nm with a mean pore size ~5 nm for electrochemically dealloyed AuAgPt (NPG-Pt).

### 3.2. Sensing Mechanism

The nanoscale nature of NPG allows for novel nanoscale transduction mechanisms to be leveraged for sensing. These transduction pathways originate from surface interactions of NPG as the concentration of volatile compounds changes. When the surrounding environment is free of volatile compounds, the surfaces of NPG ligaments are free of adsorbed VOC molecules. Volatile compounds adsorb to the ligament surfaces of NPG as the VOC concentration increases. The adsorption of VOCs proceeds initially at random sites; subsequently, monolayers and multilayers of adsorbed VOCs form. This process can be described by Brauner–Emmett–Teller (BET) theory [24]. While this process is identical to the one that occurs on a flat gold surface, the number of adsorbed molecules is significantly higher due to the high surface area of NPG.

One crucial difference from flat gold is capillary condensation—the spontaneous filling of the pores at concentrations below the saturation pressure (P_sat_). Due to the high confinement of the capillaries within nanoporous gold, the curvature of condensed liquid works to lower the thermodynamic barrier to capillary filling—allowing for condensation to occur at concentrations lower than P_sat_ [25,26,27,28].

There are two transduction routes being leveraged that affect the frequency response of NPG as the concentration of VOCs changes in the surrounding environment. The ligaments are smaller (~14 nm) than the electron mean free path in monocrystalline Au (35 nm), indicating that surface scattering events have a more dominant effect than bulk scattering events [19,22]. As the adsorption of chemicals occurs on the surface, the behavior of conduction electrons changes due to the interaction with the orbitals of the adsorbed compound and the scattering behavior at the surface changes [29,30,31]. This produces a measurable change in resistance, which can be leveraged to probe the surface coverage of NPG, providing insight into the adsorption process and by extension the surrounding environment. There exist obvious limitations to this process—as subsequent multilayers adsorb to the surface, the interaction between the surface where electrons scatter and the newly adsorbed compound becomes limited and therefore the resistance change induced becomes marginal. Adsorption of compounds with stronger interaction to the Au has been demonstrated to shift the measured resistance [30]. Resistance changes allows the sensing platform to probe both the degree of adsorption and the strength of adsorbate interaction.

The limited sensing range of resistance creates the need for an alternative route that can complement measurements of resistance changes. Given that electrical measurements are being made, a natural extension is to study the complex frequency response of NPG. Capacitance changes simultaneously occur due to changes in the dielectric medium from air to a condensed VOC and the formation of an electric double layer (EDL) at the surface as multilayers adsorb [32,33]. The EDL is a diffuse layer of ions in solution, which facilitate the storage of energy through the concentration or depletion of ions near the surface. As multiple monolayers of adsorbed molecules form on the surface, the similarity to a bulk solution allows for the development of an EDL and complex frequency responses to evolve. Due to the nature of the double layer occurring when a more fully developed liquid layer is present, it complements the limitations of the first chemoresistive mechanism. The degree to which capacitance changes provides insight into VOCs because of the change in dielectric constant and the formation of the EDL. The compounds where an EDL can form are ones which can dissociate or cause the dissociation of a gas molecule in the air stream. As shown in previous work, the EDL formed when NPG is utilized as a humidity sensor is dependent on the dissociation of carbonic acid from dissolved carbon dioxide. This occurs to different degrees in different VOCs, from some dissociation in polar VOCs, like ethanol, to no dissociation in nonpolar VOCs, like hexane [20]. Since the concentration of ions will shift the degree to which the capacitance changes via the EDL, the capacitive changes are also highly dependent on the VOC being analyzed. These dual sensitive mechanisms not only complement one another but provide an excellent two pronged approach to classification.

#### Proposed Circuit Model

**Figure 2:**
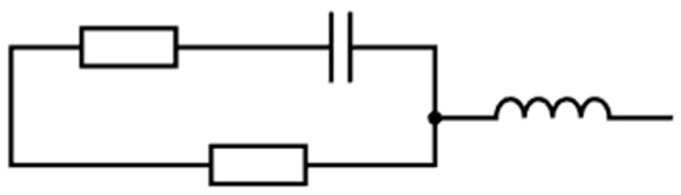
Proposed circuit model for NPG.

Based on the hypothesized transduction model, we propose a simple circuit model (Figure 2) for a NPG sensor, where the two sensitive mechanisms, represented by a resistor and capacitor, are in parallel branches to one another. These mechanisms conduct independently of one another and are therefore in parallel. An additional resistor is in series with the capacitor representing conduction between pores. A parasitic inductor is in series with the two parallel branches due to the metallic nature of NPG. This is a variation of the model of traditional parasitic effects of a resistor, but in this application, the “parasitic” capacitor is being leveraged for sensing. The variety of environmentally sensitive circuit components indicates that measuring the complete frequency response of NPG under different environments may produce greater insights into sensing.

### 3.3. Saturated Reseponse

**Figure 3:**
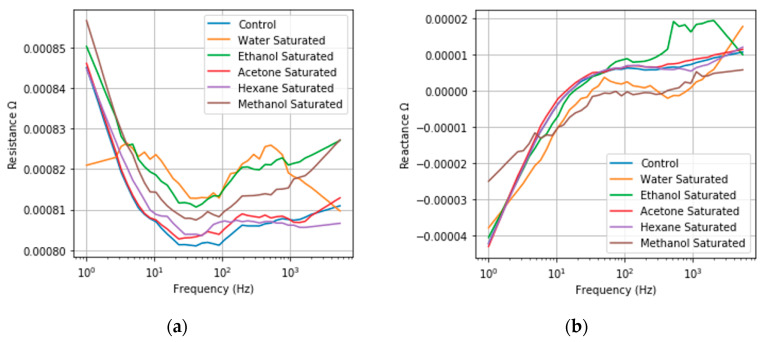
Saturated frequency responses of NPG (**a**) Resistance Frequency Response; (**b**) Imaginary Frequency Response under exposure to a saturated vapor pressure of acetone, ethanol, hexane, methanol, water, and a control in dry air. Resistance changes varied from 1 × 10^−5^ to 2.03 × 10^−5^ Ω for the different model compounds. Changes in the capacitance are observed by changes in the saturated reactance responses, via the increasing corner frequency in saturated responses relative to the dry air control. Only frequencies up to 7.5 kHz are plotted for clarity.

The frequency responses of NPG under saturated condition for the five model compounds as well as NPG under dry air are plotted in Figure 3. There are clear changes from the control in both the resistance and reactance under saturated conditions for all model compounds, indicating that both proposed sensing mechanisms are valid. The effects of electron scattering are evident when observing the resistance, which increases relative to the control for all tested model compounds. The degree of change is also in line with expectations, with larger resistance changes observed for more polar compounds. The greater the polarity, the stronger the interaction between conduction electrons and the adsorbed compound, resulting in a greater frequency of scattering events and a larger measured resistance. The effects of capacitance changes can also be observed by both the changes in reactance values and the shift in the corner frequency, the point at which capacitive power falls off. Since the capacitive behavior is in parallel to the resistance changes in the equivalent, capacitance cannot be interpreted directly. Changes in the equivalent capacitance are evidenced by the observed changes in reactance values and the shift in corner frequency.

### 3.4. Classification of Model Compounds

**Figure 4:**
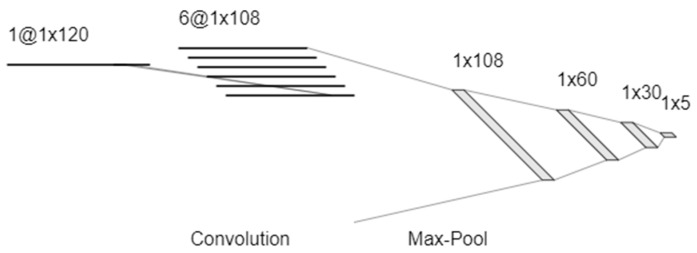
CNN Architecture for classification of the five model compounds.

While the capacitive changes manifest themselves in the recorded frequency response in different ways, the overall response as the partial pressure changes is even more complex. Previous work has shown that changes in resistance and capacitance are highly nonlinear with the partial pressure of VOCs. Due to the unique and unknown nature in which each individual model VOC will shift the frequency response, it becomes an incredibly challenging classification problem for linear data science methods. Attempting to use these classifiers (support vector machine, linear discriminant analysis, and logistics regression) yielded poor accuracy (<50%). In order to address the complex nonlinearities of the adsorption and condensation process, a neural network was used. The network architecture is detailed in Figure 4. A CNN was used in order to characterize patterns, such as the shifting corner frequency at low frequency due to the equivalent parallel RC circuit, but also other subtle patterns that may be used for classification, such as the high frequency corner created by the onset of inductive behavior.

**Figure 5:**
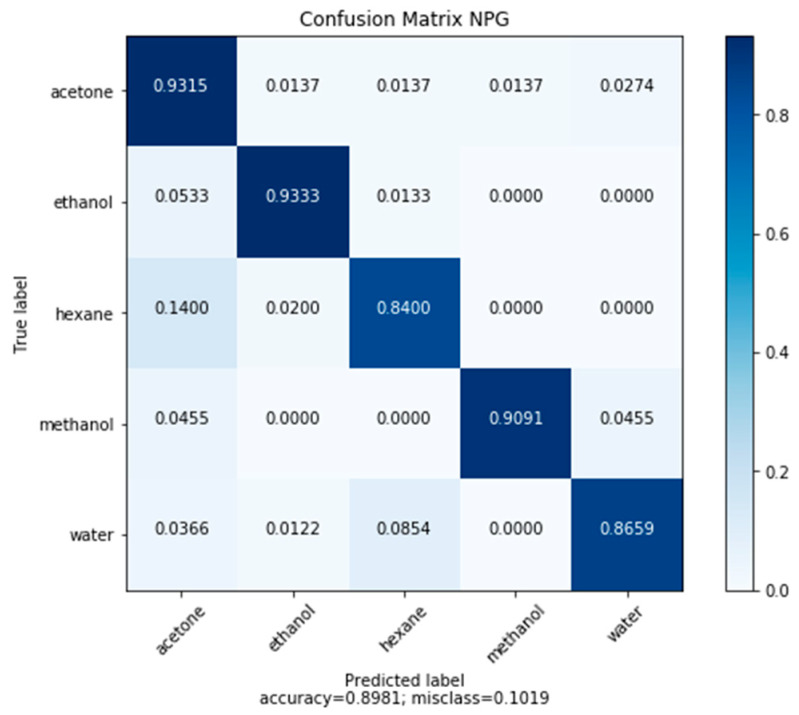
Confusion matrix of test set predictions made by the convolutional neural network (CNN) (a classification network) based on frequency response data on NPG.

In order to train the model, measurements for each of the five model compounds were taken at 10% intervals relative to P_sat_ between 0% and 100% P_sat_. These measurements were repeated five times at each concentration for each model compound. Testing was done using a 5-fold shuffle split, where a random selection of 80% of the data was used to train the model and 20% was used to evaluate the trained model. An overall accuracy of 89.81% was achieved in testing. Complete results are shown in Figure 5. The misclassifications fell into two categories, a misclassification at low concentration and misclassification of hexane. At low concentration the responses are both smaller and not able to leverage the capacitive sensitivity due to the lack of pore filling. The small response increases the likelihood that the algorithm is fitting to noise rather than the underlying signal. Hexane misclassifications can be rooted in the fact that hexane has the weakest sensitivity. Looking at the saturated response, hexane showed the smallest change from the control, making it the most likely to produce a unique signal response compared to the other compounds. This is due to the nonpolar nature of hexane producing a weak electronic interaction with the gold surface. These challenges are addressed by increases in surface area and functionalization.

### 3.5. Regression of Volatile Concentration

**Figure 6:**
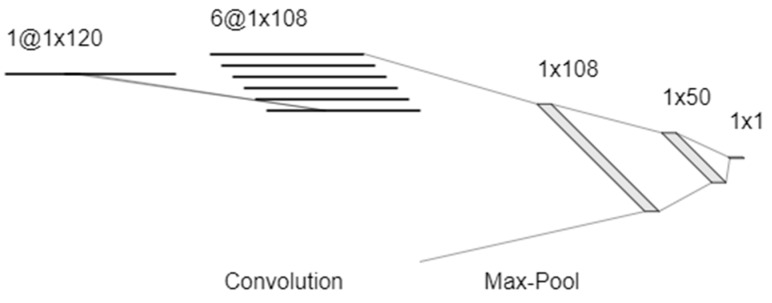
CNN architecture for regression of the five model compounds.

Due to the aforementioned nonlinearities and complexity in relation to the frequency response, a similar neural network architecture was used to build a regression model to quantify the concentration of each individual VOC. A CNN was used once again to pick up on patterns within the measured frequency response signal. The architecture of the model is shown below in Figure 6.

**Figure 7:**
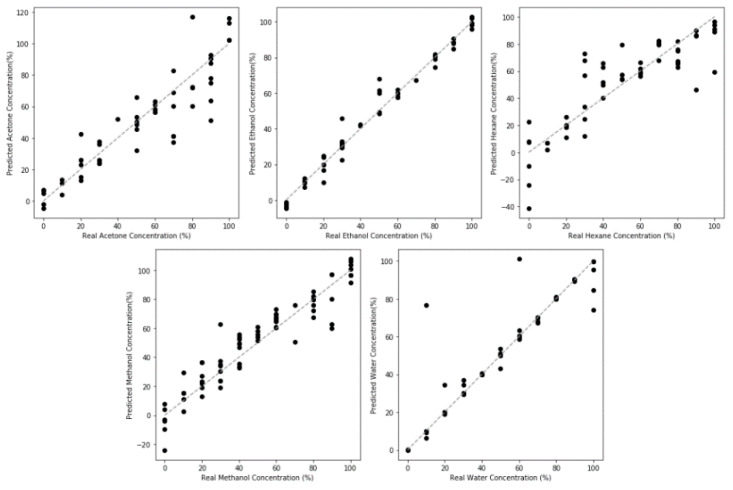
Regression model performance for NPG sensing of the five model VOCs studied. The reduced sensing performance towards nonpolar compounds, such as hexane, in comparison to polar compounds, such as water, can be seen by the smaller deviation in the response. Nonlinear bias can be observed by the skew at high concentrations.

Regression models were evaluated in a similar manner using a 5-fold shuffle split, with the standard deviation/resolution shown in Table 1. The test set results are plotted in Figure 7. Looking at the regression performance, the usage of the neural network to compensate for the nonlinear behavior of adsorption as well as the complex behavior of both resistive and capacitive changes produced nonbiased and accurate regression results through the majority of the concentration range for four of the model compounds (acetone, ethanol, methanol and water), indicating the neural network was successful when compared to simple linear methods.

The same issues that affected the classification model also affect the regression models. Performance in detecting hexane is significantly lower, with much lower R^2^ and worse resolution. This is due to the aforementioned low sensitivity to hexane. This weak interaction is underscored by the second worst performing fit, acetone. The greater the nonpolarity of the compound, the smaller the interaction and the poorer the resulting model accuracy is. Furthermore, the models performed the worst at the low concentration and near saturation regimes. As mentioned in the discussion about the classification model, the low concentration regime is inherently difficult to sense due to the small signal response produced when very few VOC molecules are present to interact with the sensor. At high concentration, the response saturates once all of the pores have been filled. With the lowering of the thermodynamic barrier to condensation, the point at which no additional pore filling occurs will also shift to lower concentrations. At this point, no additional resistance or capacitance changes can occur and this is one of the fundamental limitations of this porous sensor.

### 3.6. Sensitivity Amplification by Pt Refinement

A simple strategy in order to mitigate the effects at low concentration is to increase the surface area, thereby improving the transduction of the environmental conditions. One well explored approach to increase the surface area of NPG is to add a small Pt content to the AuAg alloy [21]. Pt acts to limit the surface diffusion of Au, refining the pore and ligament size and increasing the surface area of the resulting nanoporous structure. This pinning phenomenon results in a high surface concentration of Pt relative to the bulk [34]. Adsorption of compounds, such as water, has been shown in simulations to have a higher bonding energy, and therefore stronger interaction with Pt than Au [35]. This greater interaction energy further improves the signal for sensing purposes.

In previous work on NPG humidity sensors, it was demonstrated that by increasing the surface area through the addition of Pt content, the effect of noise was dramatically reduced. The trade-off was a minimal decrease in sensing range [20].

**Figure 8:**
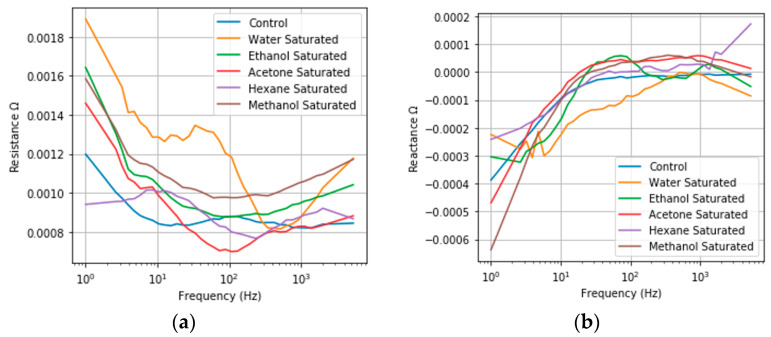
Saturated frequency responses (**a**) Resistance Frequency Response; (**b**) Imaginary Frequency Response of NPG-Pt under exposure to a saturated vapor pressure of acetone, ethanol, hexane, methanol, water and a control in dry air. An increase in the resistance response can be observed in comparison to NPG, with changes ranging from 1 × 10^−4^ to 4.03 × 10^−4^ Ω for the different model compounds. Greater shifts in the corner frequency of the reactance response are also observed.

In implementing this approach, the same general trends were observed as NPG, demonstrating consistency in physics with NPG dealloyed from AuAg. The only difference highlighted in Figure 8 is the increase in the base resistance and the increased total change when comparing the control to the saturated response, indicating a greater sensitivity.

#### 3.6.1. Classification by NPG-Pt

**Figure 9:**
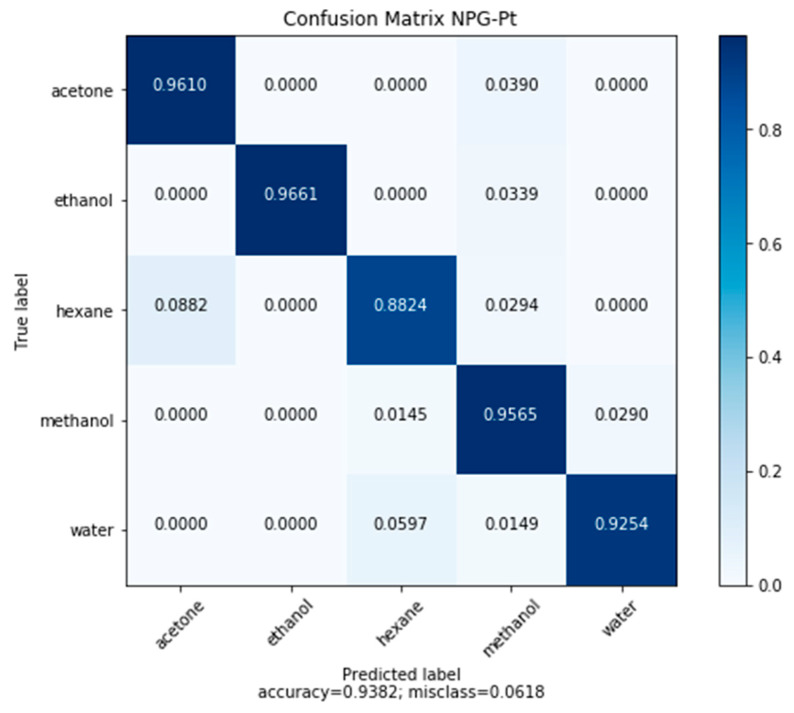
Confusion matrix of test-set predictions made by the CNN classification network based on frequency response data on NPG-Pt.

In similar classification tests utilizing the same classification neural network and training scheme, classification accuracy with NPG-Pt improved (Figure 9). A confusion matrix summarizing the results is shown in Figure 8. The results are shown below. Across the five model compounds, the number of misclassification errors at low concentration generally decreases when utilizing high surface area NPG-Pt, due to the greater sensitivity. However, increasing the surface area was not sufficient to address issues associated with the weak interaction of hexane.

#### 3.6.2. Regression Utilizing NPG-Pt Frequency Responses

**Figure 10:**
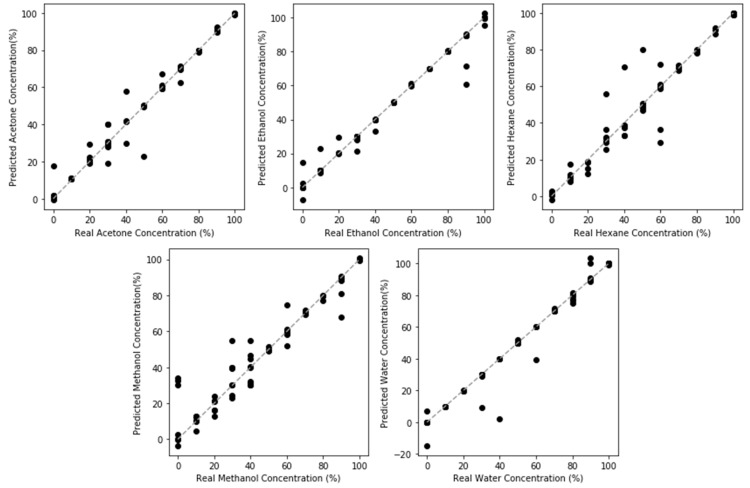
Regression Model Performance for NPG-Pt sensing of model VOCs. An improved performance compared to NPG can be observed across the model compounds, through smaller variations, due to the higher surface area of transduction.

The benefits of high surface area are also apparent in regression tasks (Figure 10). The resolution, shown in Table 2, substantially improved for all model compounds. While bias is still observed at high concentration, it is not substantially different from NPG. In comparing the performance between model compounds, the best performance was observed for the polar compounds (water, methanol) and the worst performance for the most nonpolar compound (hexane). As discussed with regards to NPG regression performance, this can be attributed to both the weaker surface adsorption interaction and the minimal capacitive changes associated with nonpolar compounds.

### 3.7. Effects of Thiol Functionalization

Nonpolar compounds make up a significant portion of widely used VOCs, particularly in the oil and gas industry, making them an important class of VOCs to detect. While NPG and NPG-Pt demonstrated nonpolar VOCs sensing, the resolution and the sensitivity can be further improved through the usage of chemical functionalization. One simple functionalization strategy is to change the nature of the NPG surface by the formation of a self-assembled thiol monolayer (SAM) on the surface. Material characterization studies of NPG and NPG-Pt done in previous work have demonstrated that the surface is rich in Au [36]. Thiols are well known to form a SAM on Au surfaces due to the strong interaction of the sulfur atom and Au atom. Accordingly, the surface of NPG-Pt was functionalized with 1-dodecanethiol (tNPG-Pt). This provides a simple method to make the surface highly hydrophobic surface, which facilitates greater adsorption and condensation of nonpolar compounds.

Rather than relying on the direct interaction between the volatile compound and the surface to induce resistance changes, thiolated NPG leverages the change in the interaction between the thiol molecule and the surface. The tight interaction between the Au and the S atom increases resistance through surface scattering, in the same way as adsorbed VOCs. As nonpolar volatile compounds condense on the surface, the condensed nonpolar VOCs solvate the thiol monolayer. This solvation reduces the surface scattering effects and therefore reduces the measured resistance. The thiolated surface also facilitates capacitive change due to the increase in the dielectric constant once solvated.

**Figure 11:**
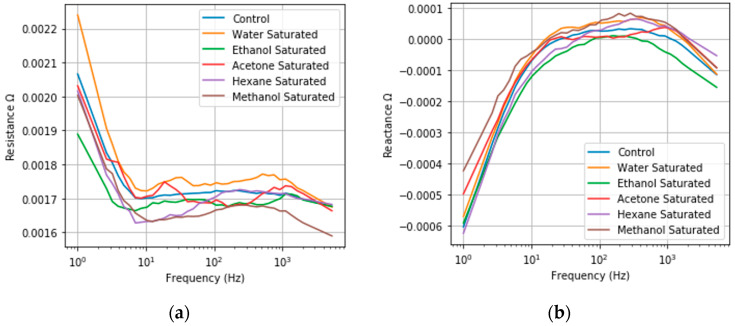
Saturated frequency responses of tNPG-Pt (**a**) Resistance Frequency Response; (**b**) Imaginary Frequency Response under exposure to a saturated vapor pressure of acetone, ethanol, hexane, methanol, water and a control in dry air. In contrast to NPG and NPG-Pt, the saturated response for the all compounds except water demonstrate a decreasing resistance relative to the dry air control, due to the indirect sensing mechanism of the thiol layer. Resistance changes vary from 0.5 × 10^−4^ Ω–1.42 Ω. Reactance changes are also observed in tNPG-Pt samples allowing for multivariate sensing.

The saturated response of tNPG-Pt is plotted in Figure 11. The interaction between the SAM and the ligament surface is manifested through the increase in the measured resistance when comparing the frequency responses of NPG-Pt and tNPG-Pt. The frequency responses of tNPG-Pt and NPG-Pt under saturated volatile conditions are notably different. For thiolated materials, there is a significant observed resistive change for all volatile compounds tested, while nonpolar compound responses shifted in the opposite direction relative to nonthiolated responses. This confirms the hypothesized mechanism, where the weakening of the Au-S bond decreases the resistance. A notable exception to this is water. Due to its polar nature, water is unable to solvate the thiol molecules and the resulting interaction does not follow the same mechanism.

#### 3.7.1. Classification of Model Compounds Utilizing tNPG-Pt Frequency Responses

**Figure 12:**
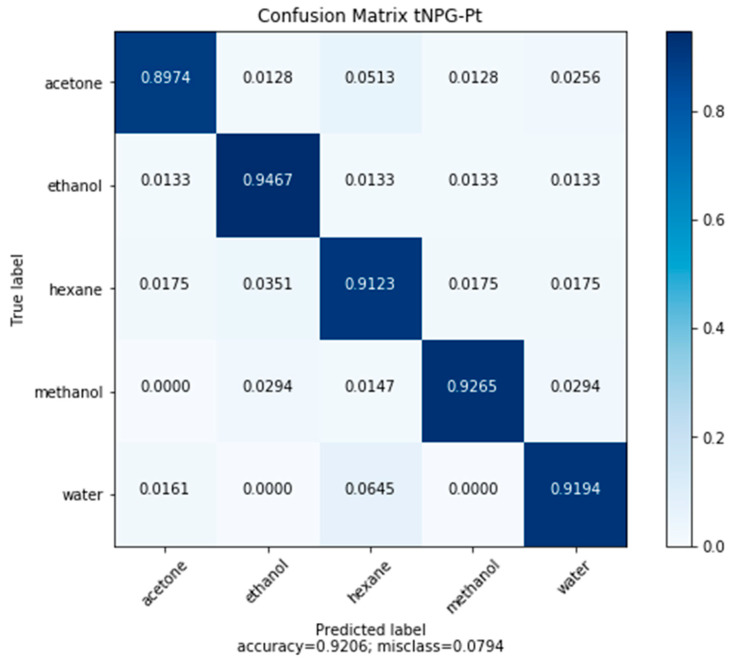
Confusion matrix of test set predictions made by the CNN classification network based on frequency response data on tNPG-Pt.

A classification CNN was fit using the aforementioned method for NPG and NPG-Pt. The results are plotted below in Figure 12. By leveraging an indirect mechanism, where the response is based on the weakened thiol-Au interaction rather than a direct interaction between nonpolar VOC and Au, the misclassification error for nonpolar compounds decreased. These misclassifications in NPG and NPT-Pt were due to the smaller effect of noise at low concentration for nonpolar compounds, such as hexane. Notably, the misclassification rate for polar compounds, such as water, rose, particularly at low concentrations. This increase in misclassification is attributed to low concentrations of water being unable to adsorb onto the functionalized hydrophobic surface of tNPG-Pt nor interact with the thiol molecules.

#### 3.7.2. Regression Utilizing tNPG-Pt Frequency Responses

**Figure 13:**
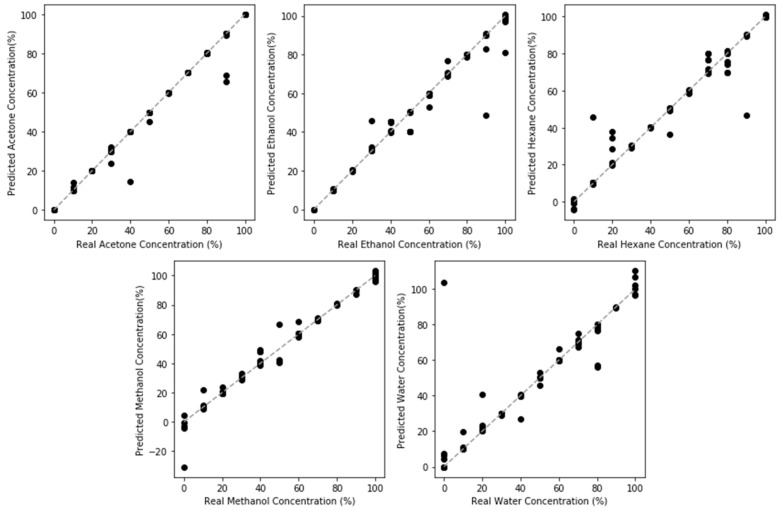
Regression Model Performance for tNPG-Pt sensing of model VOC. A significant improvement in the sensing of nonpolar compounds can be observed by the reduced variance in sensing performance, in compounds such as hexane. This performance improvement comes at the trade-off of decreased sensitivity towards polar compounds, such as water.

Through functionalization, tNPG-Pt demonstrated significant improvements in quantifying the concentration of nonpolar compounds with improvements in both the resolution and the R^2^ score, shown in Table 3. Performance of regression is plotted in Figure 13. The indirect route, where absorbed compounds influence the electrical properties by solvating the thiol monolayer, creates a stronger change in comparison to direct interaction of nonpolar compounds and the NPG-Pt surface.

These improvements in sensing nonpolar compounds come at the cost of increased bias for nonpolar compounds at high concentration. Due to the hydrophobic nature of the surface, condensation occurs, subsequently followed by saturation of nonpolar compounds at a lower partial pressure. This underscores the inherent trade off in NPG based gas sensors systems where improvements in accuracy and precision come at the cost of sensor range. While the response to water is diminished, there is still a sensitivity to water. This is because thiol layers do allow for water adsorption and condensation at high vapor concentrations. Water adsorption can initiate at boundaries where the thiol layer is disrupted, which are more common due to the curvature of NPG-Pt in comparison to flat gold; this adsorption facilitates some, but reduced sensing ability.

## 4. Conclusions

In conclusion, this work accomplished a variety of sensing tasks.

1. Convolutional neural networks were used to overcome the issues of nonlinearity and complex response behavior for the class of NPG sensors
2. NPG demonstrated ability to classify and quantify five model volatile organic compounds (acetone, ethanol, hexane, methanol, water) through measurements of the frequency response of nanoporous gold, with an accuracy of 89.81%.
3. CNN models produced nonbiased accurate quantification of the model compounds, except for hexane. The poorest performance occurred at low concentrations across all model compounds.
4. Classification accuracy was improved by refining the pore size using NPG-Pt (93.82%). Regression performance for all model compounds improved at low concentrations.
5. tNPG-Pt demonstrated an improved response for nonpolar compounds tested, with classification accuracy of hexane improving from 88.24% to 91.23%.
6. Furthermore, regression of hexane concentration improved substantially.
7. Improvements in sensing hexane came with a trade-off in water sensitivity and selectivity.

This study demonstrates the potential of NPG sensors for sensing volatile organic compounds. While only five model compounds were tested, this could be expanded substantially to a broader range of VOCs. Furthermore, the empirical results demonstrate that functionalization of the surface can tune the sensitivity and selectivity of the sensor, allowing for an infinite iteration space through control of the material properties.

## Figures and Tables

**Table 1:** Summary of metrics of regression across five model volatile organic compounds (VOCs) for NPG.

Compound	R^2^	Standard Deviation (P/P_sat_%)
Acetone	0.764	15.6
Ethanol	0.971	5.2
Hexane	0.724	16.4
Methanol	0.884	10.4
Water	0.892	10.6

**Table 2:** Summary of metrics of regression across five model VOCs for NPG-Pt.

Compound	R^2^	Standard Deviation (P/P_sat_%)
Acetone	0.961	5.4
Ethanol	0.974	5.1
Hexane	0.932	8.0
Methanol	0.902	9.2
Water	0.963	6.4

**Table 3:** Summary of metrics of regression across five model VOCs for tNPG-Pt.

Compound	R^2^	Standard Deviation (P/P_sat_%)
Acetone	0.975	4.6
Ethanol	0.960	6.1
Hexane	0.948	7.5
Methanol	0.972	5.0
Water	0.963	6.4
